# Coherent Integration Method Based on Radon-NUFFT for Moving Target Detection Using Frequency Agile Radar

**DOI:** 10.3390/s20082176

**Published:** 2020-04-12

**Authors:** Jiameng Pan, Qian Zhu, Qinglong Bao, Zengping Chen

**Affiliations:** 1 National Key Laboratory of Science and Technology on ATR, National University of Defense Technology, Changsha 410073, China; zhuqian10@nudt.edu.cn (Q.Z.); baoqinglong06@nudt.edu.cn (Q.B.); 2 School of Electronics and Communication Engineering, Sun Yat-sen University, Guangzhou 510275, China; chenzengp@mail.sysu.edu.cn

**Keywords:** frequency agile radar, moving target detection, coherent integration, nonuniform fast Fourier transform

## Abstract

This paper considers the coherent integration problem for moving target detection using frequency agile (FA) radar, involving range cell migration (RCM) and the nonuniform phase fluctuations among different pulses caused by range-agile frequency (R-AF) coupling and velocity-time-agile frequency (V-T-AF) coupling. After the analysis of the term corresponding to the phase fluctuation caused by V-T-AF coupling, the term can be regarded as related to an equivalent non-uniform slow time, and nonuniform fast Fourier transform (NUFFT) could be the solution. So a fast coherent integration method combining Radon Fourier transform (RFT) and NUFFT based on low-rank approximation, i.e., Radon-NUFFT, is proposed. In this method, the RCM is solved by Radon algorithm via target trajectory searching, the non-uniform phase fluctuation caused by R-AF coupling is compensated by constructing a compensation item corresponding to the range and agile frequency. In addition, the compensation of the non-uniform phase fluctuation caused by V-T-AF coupling is converted into a problem of spectral analysis of non-uniform sampling complex-valued signal, which is solved by the NUFFT based on low rank approximation. Compared with the existing methods, the proposed method can realize the coherent integration for FA radar accurately and quickly. The effectiveness of the proposed method is verified by simulation experiments.

## 1. Introduction

In modern warfare, the capability of anti-jamming is critical to radar system for the survivability in battlefield. Radar that incorporate frequency agility, especially pulse-to-pulse carrier frequency agility, has the advantages of enhanced jam resistance and low probability of detection and interception [1]. In addition, frequency agile (FA) radar can also improve the range resolution because of the large synthetic bandwidth [2]. Therefore, there has been a renewed interest in FA radar in recent years.

In FA radar using waveform with pulse-to-pulse carrier frequency agility, the center frequency of each transmitted pulse is hopped randomly between certain frequency points, which demolishes the coherence between pulse-to-pulse radar echo. However, coherent integration is an effective anti-clutter technique for radar, and it enhances the capability of moving target detection since it provides the largest signal-to-noise (SNR) output in white Gauss noise. The existing coherent integration methods, such as Keystone transform [3], Radon Fourier transform (RFT) [4], sparse discrete fractional Fourier transform [5], discrete polynomial-phase transform [6] and Radon-Lv’s Distribution [7] are only applicable for conventional radar. Also, some methods have been proposed for coherent integration of FA radar. Methods based on compressed sensing [8,9] has been applied for random carrier frequency signal, which only perform well in high SNR scenario, and the performance degrades significantly in low SNR scenario. The method in Reference [10] combines the phase compensation and cost function based on minimum entropy for coherent integration using FA radar, but the problem of range cell migration (RCM) is not discussed. Wang, et al. proposed a frequency agile coherent Radon transform (FA-CRT) method [11], which is realized by CRT among pulses combining with target trajectory searching, but it has high computational complexity due to the time-consuming searching process and compensation.

Motivated by previous work, after analyzing the signal model of moving target echo signal of FA radar, it can be concluded that there are three problems to be solved, the problem of RCM caused by target motion, the nonuniform phase fluctuations among different pulses caused by range-agile frequency (R-AF) coupling, and the nonuniform phase fluctuations caused by velocity-time-agile frequency(V-T-AF) coupling. The problem of RCM can be solved by target trajectory searching, and the phase fluctuation caused by R-AF coupling can be compensated directly at one time, the most important is the phase fluctuation caused by V-T-AF coupling. After the analysis of the term corresponding to phase fluctuation caused by V-T-AF coupling, the term an be regarded as related to a equivalent non-uniform slow time, so the problem can be converted to a problem of spectral analysis of non-uniform sampling complex-valued signal. As for spectral analysis of nonuniformly sampled data, method based on Lomb-Scargle periodogram (LSP) are not applicable for complex-valued data [12], and method based on iterative adaptive approach (IAA) requires a lot of iterative calculations [13]. So we adopt nonuniform discrete Fourier transform (NUDFT) for the spectral analysis of nonuniformly sampled data [14]. Fast algorithms for computing the NUDFT are referred to as nonuniform fast Fourier transform (NUFFT), and state-of-the-art NUFFT algorithms are usually based on oversampling and interpolation [15,16,17], min-max interpolation [18], and low-rank approximation [19]. Since the equivalent non-uniform slow time corresponding to the V-T-AF coupling term is approximately equispaced, and the NUFFT method based on low-rank approximation is suitable for that condition, so we adopt NUFFT based on low-rank approximation to deal with the V-T-AF coupling.

The remainder of this paper is organized as follows. Section 2 establishes the signal model of echo signal and the problems for coherent integration of FA radar are raised. In Section 3, the coherent integration method based on Radon-NUFFT is described in detail. Section 4 presents the simulation results to verify the performance of the proposed method. Section 5 concludes the paper.

## 2. Signal Model

**Figure 1:**
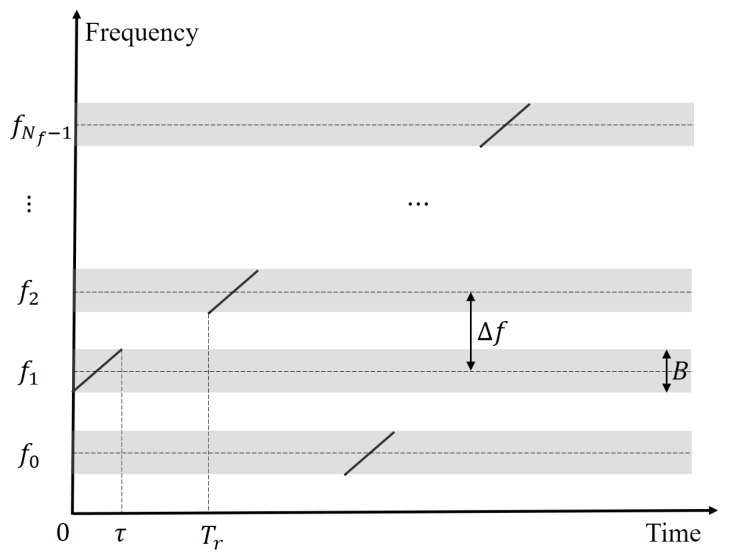
Simplified diagram of the transmitted signal.

Assume the FA radar adopts linear frequency-modulated (LFM) signal as the baseband waveform, where the carrier frequency of each pulses varies randomly between a fixed set of frequency points evenly distributed in a fixed working band. The set of fixed frequency points is $F=\{f_{i}|f_{i}=f_{0}+i\Delta f,i=0,1,\cdots ,N_{f}-1\}$, where $f_{0}$ is nominal frequency, Δf is the frequency interval between two adjacent frequency points, and $N_{f}$ is the total number of frequency points. The transmitted signal can be expressed as

(1) $$s_{t}(t_{m},\hat{t})=A\mathrm{Rect}\left(\frac{\hat{t}}{\tau }\right)\exp\left[j2\pi f_{m}\left(t_{m}+\hat{t}\right)\right]\exp\left(j\pi \mu {\hat{t}}^{2}\right)$$

where $t_{m}=mT_{r}$ is the slow time, *m* indicates the pulse number, $T_{r}$ is the pulse repetition interval (PRI), and $\hat{t}$ is the fast time. $\mathrm{Rect}\left(x\right)=\left\{\begin{array}{cc} 1 & \mathrm{if}\;0\leq x\leq 1,\\ 0 & \mathrm{if}\;x>1. \end{array}\right.$ is the window function, τ is the pulse width, μ=B/τ is the chirp rate of the LFM signal with bandwidth *B*, $f_{m}$ is the carrier frequency of the *m*th pulse, $f_{m}=f_{0}+c_{m}\Delta f$, where $c_{m}$ is a random integer in the range of $\left[0,N_{f}-1\right]$. Figure 1 is a simplified diagram of the time-frequency diagram of the transmitted signal, where the carrier frequency of each pulse hops randomly between different frequency points.

Suppose there is a target moving towards the radar with a constant radial velocity $v_{0}$, and the initial radial range is $r_{0}$, so the distance between the target and radar can be expressed as

(2) $$r\left(t_{m},t\right)=r_{0}-v_{0}\left(t_{m}+\hat{t}\right)$$

Therefore, the the echo signal scattered from the target received by the radar can be represented as

(3) $$\begin{array}{cc} s_{r}(t_{m},\hat{t})= & \sigma_{r}A\mathrm{Rect}\left(\frac{\hat{t}-2r(t_{m},t)/c}{\tau }\right)\exp\left[j2\pi f_{m}\left(t_{m}+\hat{t}-2\frac{r(t_{m},t)}{c}\right)\right]\\ \; & \cdot \exp\left[j\pi \mu {\left(\hat{t}-2\frac{r(t_{m},t)}{c}\right)}^{2}\right] \end{array}$$

where $\sigma_{r}$ is the scattering coefficient of target, *c* is the speed of light. Since the waveform of transmitted signal is transparent to the receiver, the baseband signal after down-conversion can be expressed as

(4) $$\begin{array}{cc} s_{b}(t_{m},\hat{t})= & \sigma_{r}A\mathrm{Rect}\left(\frac{\hat{t}-2r(t_{m},t)/c}{\tau }\right)\exp\left[j2\pi f_{m}\left(\hat{t}-2\frac{r(t_{m},t)}{c}\right)\right]\\ \; & \cdot \exp\left[j\pi \mu {\left(\hat{t}-2\frac{r(t_{m},t)}{c}\right)}^{2}\right] \end{array}$$

Then we can construct baseband reference signal for each echo signal, for example, the *m*th reference signal is as follows

(5) $$s_{ref}\left(t\right)=A\mathrm{Rect}\left(\frac{t}{\tau }\right)\exp\left[j2\pi (f_{m}t+\frac{1}{2}\mu t^{2})\right]$$

Using the baseband reference signal $s_{ref}\left(t\right)$ to perform matched filtering on the baseband echo signal $s_{b}(t_{m},\hat{t})$, i.e., they perform cross-correlation between them, and the result can be expressed as

(6) $$\begin{array}{cc} s_{pc}(t_{m},\hat{t})= & \sigma_{r}A\sqrt{B\tau }\mathrm{sinc}\left[\pi B\left(\hat{t}-\frac{2\left(r_{0}-v_{0}t_{m}\right)}{c}+\frac{2v_{0}f_{m}}{\mu c}\right)\right]\\ \; & \cdot \exp\left[-j2\pi \left(f_{m}-\frac{2v_{0}f_{m}}{c}\right)\cdot \frac{2\left(r_{0}-v_{0}t_{m}\right)}{c}\right] \end{array}$$

It can be seen that the signal envelope varies with $t_{m}$, and there is an offset of $2v_{0}f_{m}/\mu c$ from the correct position of the target, which is the range-Doppler coupling effect. It can be seen that the range Doppler coupling of different pulses will cause RCM only if the following condition is met

(7) $$\left[\max\left(\frac{2v_{0}f_{m}}{\mu c}\right)-\min\left(\frac{2v_{0}f_{m}}{\mu c}\right)\right]/\mu c>\frac{1}{f_{s}}$$

where $f_{s}$ is the sampling rate, Equation (7)can be expressed as

(8) $$\frac{2v_{0}N_{f}\Delta f}{\mu c}>\frac{1}{f_{s}}$$

According to the parameter of FA radar in reality and the speed of conventional air target, the quantitative analysis of Equation (8) is carried out, and it can be concluded that this situation will not be satisfied in general. It can be considered that the range-Doppler coupling effect of different pulses is the same, so this item can be ignored. In addition, since $|v_{0}|<<c$, we can get $f_{m}-2v_{0}f_{m}/c\approx f_{m}$. So Equation (6) can be simplified as

(9) $$\begin{array}{cc} s_{pc}(t_{m},\hat{t})= & \sigma_{r}A\sqrt{B\tau }\mathrm{sinc}\left[\pi B\left(\hat{t}-\frac{2\left(r_{0}-v_{0}t_{m}\right)}{c}\right)\right]\cdot \exp\left[-j2\pi \left(f_{0}+c_{m}\Delta f\right)\cdot \frac{2\left(r_{0}-v_{0}t_{m}\right)}{c}\right]\\ = & \sigma_{r}A\sqrt{B\tau }\mathrm{sinc}\left[\pi B\left(\hat{t}-\frac{2\left(r_{0}-v_{0}t_{m}\right)}{c}\right)\right]\cdot \exp\left(-j4\pi f_{0}\cdot \frac{r_{0}}{c}\right)\\ \; & \cdot \exp\left(-j4\pi c_{m}\Delta f\cdot \frac{r_{0}}{c}\right)\cdot \exp\left[j4\pi \left(f_{0}+c_{m}\Delta f\right)\cdot \frac{v_{0}t_{m}}{c}\right] \end{array}$$

It can be seen that the peak position of the signal envelope is $2(r_{0}-v_{0}t_{m})/c$. When the RCM occurs, it needs to be compensated to ensure the performance of coherent integration. Besides, it can be seen that the phase of the signal with respect to the slow time can be decomposed to two terms, the first term is $\phi_{1}=\exp\left(-j4\pi c_{m}\Delta f\cdot r_{0}/c\right)$, and the second term is $\phi_{2}=\exp\left[j4\pi \left(f_{0}+c_{m}\Delta f\right)\cdot v_{0}t_{m}/c\right]$. The first term is the nonuniform phase fluctuation caused by the coupling of target distance and the agile frequency, which is denoted as R-AF coupling. The second term is the nonuniform phase fluctuation caused by the coupling of the velocity of target and the changing slow time and agile frequency, which is denoted as V-T-AF coupling, rewrite $\phi_{2}$ as

(10) $$\phi_{2}=\exp\left[j4\pi \left(f_{0}+c_{m}\Delta f\right)\cdot \frac{v_{0}t_{m}}{c}\right]=\exp\left[j4\pi \frac{v_{0}}{c}f_{0}mT_{r}\left(1+\frac{c_{m}\Delta f}{f_{0}}\right)\right]$$

Therefore, $\phi_{2}$ can be regarded as a term related to the non-uniform slow time ${\tilde{t}}_{m}=mT_{r}\left(1+c_{m}\Delta f/f_{0}\right)$, so the compensation of V-T-AF coupling can be regarded as a problem of spectral analysis of non-uniform sampling signal.

## 3. Proposed Method

In this section, we will propose a coherent integration method based on Radon-NUFFT to solve the problems raised in Section 2. According to the previous analysis, the problems in coherent integration for FA radar are the RCM and phase fluctuations caused by R-AF coupling and V-T-AF coupling. First, we can refer to the RFT algorithm to use Radon algorithm via target trajectory searching to compensate the RCM after compensation of R-AF coupling. Then, according to the form of $\phi_{2}$ in Equation (10), we can consider it as signal sampled at non-uniform time ${\tilde{t}}_{m}$. Therefore, the accumulation of the signal is transformed into the problem of spectral analysis of the non-uniform sampling signal, so we use NUFFT algorithm to deal with it. As the frequency variation range of the FA radar is relatively small compared with the nominal frequency $f_{0}$, it can be seen from the form of ${\tilde{t}}_{m}=mT_{r}\left(1+c_{m}\Delta f/f_{0}\right)$ that the sample points are approximately equispaced, so we adopt a NUFFT algorithm based on low rank approximation.We first introduce the NUFFT algorithm based on low rank approximation, and then the coherent integration method based on Radon-NUFFT is described in detail.

### 3.1. NUFFT Based on Low Rank Approximation

NUFFT is a fast implementation of NUDFT, so we will introduce NUDFT first. Let N>1 be an integer and $x={\left(x_{0},\cdots ,x_{N-1}\right)}^{T}$ is a N×1 complex vector, the NUDFT transforms x into another N×1 complex vector $y={\left(y_{0},\cdots ,y_{N-1}\right)}^{T}$, which is defined as

(11) $$y_{n}=\sum_{k=0}^{N-1}x_{k}e^{-j2\pi t_{k}\omega_{n}},0\leq n\leq N-1$$

where $t_{0},\cdots ,t_{N-1}\in \left[0,1\right]$ are nonuniform sample points and $\omega_{0},\cdots ,\omega_{N-1}\in \left[0,N\right]$ are frequencies. We only research the situation that the frequencies are uniform, i.e., $\omega_{n}=n$, so the problem is referred as NUDFT in type II (NUDFT-II) [17].

A convenient and compact way to write Equation (11) is as a matrix-vector product, given $x\in C^{N\times 1}$, compute $y\in C^{N\times 1}$ as:

(12) $$y=\tilde{F_{2}}x,{\left(\tilde{F_{2}}\right)}_{nk}=e^{-j2\pi nt_{k}},0\leq n,k\leq N-1$$

Therefore, the problem is simply a quasi optimal complexity algorithm for computing the matrix-vector product $\tilde{F_{2}}x$. In conventional DFT where $t_{k}=k/N$, we use the notation $F_{nk}=e^{-j2\pi nk/N}$ for the DFT matrix, and FFT algorithm computes Fx in O(NlogN) operations by exploiting algebraic redundancies [20]. However, the ideas behind the FFT are not useful when the sample points are nonuniform.

While a naive application of Equation (12) results in an $O(N^{2})$ algorithm for computing the NUDFT, a fast algorithm based on FFT is referred to as NUFFT. In general, NUFFT leverage the FFT by converting the nonuniform problem into a uniform problem (or a sequence of uniform problems) to which the FFT can be applied.

Suppose that sample points $t_{0},\cdots ,t_{N-1}$ are near-equispaced, so that there exists a parameter 0<γ≤1/2 satisfying

(13) $$\left|t_{k}-\frac{k}{N}\right|\leq \frac{\gamma }{N}$$

This assumption guarantees that the closest uniform point to $t_{k}$ is k/N. Since the frequencies are uniform, i.e., $\omega_{n}=n,0\leq n\leq N-1$, we can factor the entries of $\tilde{F_{2}}$ as

(14) $${\left(\tilde{F_{2}}\right)}_{nk}=e^{-j2\pi nt_{k}}=e^{-j2\pi n(t_{k}-k/N)}e^{-j2\pi nk/N},0\leq n,k\leq N-1$$

which shows that the (n,k) entry of $\tilde{F_{2}}$ can be written as a complex number multiplied by the (n,k) entry of the DFT matrix *F*. So we can decompose $\tilde{F_{2}}$ as

(15) $$\tilde{F_{2}}=A\circ F,A_{nk}=e^{-j2\pi n(t_{k}-k/N)}$$

where ∘ is the Hadamard product.

The NUFFT algorithm is based on the simple observation that if the sample points are near-equispaced, then $A=\tilde{F_{2}}⊘F$ can be well-approximated by a low rank matrix [21], where ⊘ denotes the Hadamard division. That is to say, for a small integer *K*, we find that:

(16) $$A\approx A_{K}=\left(\left(u_{0}v_{0}^{T}+\cdots +u_{K-1}v_{K-1}^{T}\right)\right),u_{0},\cdots ,u_{K-1},v_{0},\cdots ,v_{K-1}\in C^{N\times 1}$$

So we have

(17) $$\tilde{F_{2}}x=\left(A\circ F\right)x\approx \left(\left(u_{0}v_{0}^{T}+\cdots +u_{K-1}v_{K-1}^{T}\right)\circ F\right)x=\sum_{r=0}^{K-1}D_{u_{r}}FD_{v_{r}}x$$

where $D_{u_{r}}=\mathrm{diag}\left({\left(u_{r}\right)}_{1},\cdots ,{\left(u_{r}\right)}_{N}\right)$. Since we can calculate $D_{v_{r}}x$ with *N* multiplications, then calculate $FD_{v_{r}}x$ through conventional FFT, and finally calculate $D_{u_{r}}FD_{v_{r}}x$ with *N* multiplications again. Therefore, an approximation to $\tilde{F_{2}}x$ can be computed in O(KNlogN) operations via K diagonally scaled FFTs. Moreover, each matrix-vector product in Equation (17) can be computed independently. All that remains is to show that A can be well-approximated by a low rank matrix $A_{K}$, then select the integer *K* and compute the vectors $u_{0},\cdots ,u_{K-1},v_{0},\cdots ,v_{K-1}$.

We can derive a low rank approximation for *A* by using Chebyshev expansions [22]. Define 0<ϵ<1 as the working precision. For an integer p≥0, the Chebyshev polynomial of degree *p* is given by $T_{p}\left(x\right)=$$\cos\left(p\cos^{-1}x\right)$ on x∈[−1,1]. If γ>0, then for 0<ϵ<1 we can find an integer *K* and a matrix $A_{K}$ satisfying ${∥A-A_{K}∥}_{\max}\leq \epsilon ,$ where ${∥\cdot ∥}_{\max}$ denotes the absolute maximum matrix entry. Define $t={\left(t_{0},\cdots ,t_{N-1}\right)}^{T}$, $e={\left(0,1/N,\cdots ,\left(N-1\right)/N\right)}^{T}$, $f={\left(0,1,\cdots ,N-1\right)}^{T}$, then the matrix $A_{K}$ can be given as

(18) $$A_{K}=\sum_{r=0}^{K-1}\left[\sum_{p=0}^{K-1}a_{pr}\left(\exp\left(-j\pi N\left(t-e\right)\right)\circ T_{p}\left(\frac{N(t-e)}{\gamma }\right)\right)\right]T_{r}\left(\frac{2f^{T}}{N}-1\right)$$

So we can define $u_{r}$ and $v_{r}$ as

(19) $$\begin{array}{cc} u_{r} & =\left[\sum_{p=0}^{K-1}a_{pr}\left(\exp\left(-j\pi N\left(t-e\right)\right)\circ T_{p}\left(\frac{N(t-e)}{\gamma }\right)\right)\right] \end{array}$$

(20) $$\begin{array}{cc} v_{r} & =\left\{\begin{array}{cc} T_{r}\left(\frac{2f}{N}-1\right)/2 & r=0\\ T_{r}\left(\frac{2f}{N}-1\right) & r\geq 1 \end{array}\right. \end{array}$$

Therefore, $A_{K}$ can also be expressed as the form in Equation (16), for 0≤p,q≤K−1, the coefficients $a_{pr}$ is defined as

(21) $$a_{pr}=\left\{\begin{array}{cc} 4j^{r}J_{(p+r)/2}\left(-\gamma \pi /2\right)J_{(r-p)/2}\left(-\gamma \pi /2\right), & \;\mathrm{mod}\;(|p-r|,2)=0\\ 0, & \;\mathrm{otherwise}\; \end{array}\right.$$

where $J_{\nu }\left(z\right)$ is the Bessel function of parameter ν at *z*.

The expansion in Equation (18) provides us with a rank *K* matrix $A_{K}$ that approximates *A* as $A=\lim_{K\to \infty }A_{K}$. Since Chebyshev expansion is convergent, for any fixed *K*, there is an explicit upper bound for $||A-A_{K}{\left|\right|}_{max}$. For γ>0, the integer *K* is given by

(22) $$K=\max\left\{3,\left\lceil 5\gamma e^{W(\log(140/\epsilon )/(5\gamma ))}\right\rceil \right\}=O\left(\frac{\log(1/\epsilon )}{\log\log(1/\epsilon )}\right)$$

where W(x) is the Lambert-W function, and ⌈x⌉ is the nearest integer above or equal to x≥0. By asymptotic approximations of W(x) as x→∞, we find that when ϵ→0, K=O(log(1/ϵ)/loglog(1/ϵ)), therefore, $\tilde{F_{2}}x$ can be computed in a total of O(NlogNlog(1/ϵ)/loglog(1/ϵ)) operations using Equation (17). In practical application, we do not need to calculate the value of *K*, but adopt the empirical value. The specific value of *K* depends on γ and ϵ, for example, when $\frac{1}{4}<\gamma \leq \frac{1}{2}$ and $\epsilon \approx 1.2\times 10^{-7}$, we can set the value as K=10.

It should be noted that the Chebyshev expansions requires $O(K^{2}N)$ operations [23] , which should be included in the final complexity of the NUFFT. However, for a batch of data with the same sampling mode, this calculation only needs to be carried out once, and $A_{K}$ is independent from the actual sampling result x, so when the NUFFT is applied to the coherent integration of FA radar, this operation only needs to be carried out once for the data in each coherent process interval (CPI). The final spectral estimate of nonuniformly sampled data sequence x at frequency $\omega_{n}$ in Equation (11) can be denoted as $y_{n}=\mathrm{NUFFT}\left(x,\omega_{n}\right)$.

### 3.2. Radon-NUFFT Method

Based on the above-mentioned analysis, we propose the Radon-NUFFT method to achieve the coherent integration for FA radar. At first, assuming that the transmitted signal is conventional LFM signal with a fixed carrier frequency $f_{c}$, which is noted as $h_{t}\left(t_{m},\hat{t}\right)$, and the echo signal after down conversion and matched filtering is $h_{pc}\left(t_{m},\hat{t}\right)$, so the we can perform coherent integration method based on conventional RFT algorithm [4], which is is expressed as

(23) $$G\left(r,v\right)=\int h_{pc}\left(t_{m},\frac{2(r-vt_{m})}{c}\right)\exp\left(-j\frac{4\pi vt_{m}}{\lambda }\right)dt_{m}$$

where *r* and *v* are the range and velocity of target, $\lambda =c/f_{c}$ is the wavelength of the signal. The implementation of RFT is to search different speed *v* and range *r* to obtain different target trajectory, and then to accumulate the energy on the trajectory through FFT algorithm.

However, for FA radar signal, due to the nonuniform phase fluctuation caused by the R-AF coupling and V-T-AF coupling, conventional FFT algorithm cannot be applied. So the principle of the coherent integration method for FA radar is defined as

(24) $$G_{RNFT}\left(r,v\right)=\int s_{pc}\left(t_{m},\frac{2(r-vt_{m})}{c}\right)\exp\left(j\frac{4\pi rc_{m}\Delta f}{c}\right)\exp\left(-j\frac{4\pi vt_{m}\left(f_{0}+c_{m}\Delta f\right))}{c}\right)dt_{m}$$

First, the coherent processing interval $T_{M}=MT_{r}$ can be preset according to radar parameters and dwell time of antenna beam, where *M* is the number of pulses. So we can get the discrete form of continuous signal $s_{pc}(t_{m},\hat{t})$, which is expressed as

(25) $$S_{pc}\left(m,n\right),\;m=1,\cdots ,M,\;n=1,\cdots ,N$$

where $N=T_{r}*f_{s}$ is the number of sampling points in fast time dimension.

The scope of velocity $\left[-v_{max},v_{max}\right]$ and scope of range $\left[r_{min},r_{max}\right]$ are determined by the moving status of targets to be detected. The interval of velocity is set as $\Delta v=c/(2Mf_{0}T_{r})$, and the interval of range is $\Delta r=c/2f_{s}$. So the number of searching velocity and range are $N_{v}=\left\lceil 2v_{max}/\Delta v\right\rceil ,N_{r}=\left\lceil \left(r_{max}-r_{min}\right)/\Delta r\right\rceil$. So the searching parameters can be defined as

(26) $$\begin{array}{cc} r_{i} & =r_{min}+\left(i-1\right)\Delta r,i=1,2,\cdots ,N_{r}\\ v_{k} & =-v_{max}+\left(k-1\right)\Delta v,k=1,2,\cdots ,N_{v} \end{array}$$

So the discrete form of Equation (24) can be expressed as follows

(27) $$\begin{array}{cc} G_{RNFT}\left(i,k\right)= & \sum_{m=1}^{M}S_{pc}\left[m,\mathrm{round}\left(\frac{2(r_{i}-v_{k}mT_{r})}{c}\right)\right]\\ \; & \cdot \exp\left(j\frac{4\pi r_{i}c_{m}\Delta f}{c}\right)\exp\left[-j\frac{4\pi v_{k}mT_{r}\left(f_{0}+c_{m}\Delta f\right)}{c}\right] \end{array}$$

We can search all the motion parameters and calculate $G_{RNFT}\left(i,k\right)$ directly as Equation (27), which is the implementation method of FA-CRT [11] algorithm. However, it requires a lot of calculations, which is not applicable in engineering application. So we need to adopt a more efficient implementation method, which is the Radon-NUFFT method. The specific process of this method is as follows.

First, for the M×N dimensional matrix $S_{pc}$, the phase fluctuation caused by the R-AF coupling should be compensated for the whole data matrix, the compensation term is given as

(28) $$C_{R}\left(m,n\right)=\exp\left(j2\pi c_{m}\Delta f\frac{n}{f_{s}}\right)$$

Each entry of $S_{pc}$, noted as $S_{pc}\left(m,n\right)$, is multiplied by the corresponding compensation term $C_{R}\left(m,n\right)$, so a new matrix ${\tilde{S}}_{pc}$ after compensation of R-AF coupling can be expressed as

(29) $${\tilde{S}}_{pc}=S_{pc}\circ C_{R}$$

Then, for a certain pair of motion parameters $\left(r_{i},v_{k}\right)$, a moving trajectory of the target is determined by the parameters, and the M×1 dimension data vector $X_{M}$ is extracted, which is defined as

(30) $$X_{M}\left(m\right)={\tilde{S}}_{pc}\left[m,\mathrm{round}\left(\frac{2(r_{i}-v_{k}mT_{r})}{c}\right)\right]$$

In the case of high-speed target and low pulse repetition frequency (PRF), it is highly possible that the Doppler frequency ambiguity would occur. Therefore, when Doppler frequency ambiguity occurs, NUFFT should be performed after compensation of the corresponding Doppler frequency ambiguity. The Doppler ambiguity factor is $K=\mathrm{round}(2v_{k}f_{0}T_{r}/c)$, so the compensation item corresponding to $X_{M}\left(m\right)$ is

(31) $$C_{A}\left(m\right)=\exp\left[-j2\pi Km\left(1+\frac{c_{m}\Delta f}{f_{0}}\right)\right]$$

So the M×1 dimension data vector after the compensation of Doppler ambiguity can be expressed as $X_{M}\circ C_{A}$, then we can perform NUFFT on $X_{M}\circ C_{A}$ at frequency point $\omega_{k}=2v_{k}f_{0}/c-K/T_{r}$ in respect to $v_{k}$ to compensate the V-T-AF coupling, which is denoted as

(32) $$G_{RFNT}\left(i,k\right)=\mathrm{NUFFT}\left(X_{M}\circ C_{A},2v_{k}f_{0}/c-K/T_{r}\right)$$

where $G_{RFNT}\left(i,k\right)$ is the coherent integration result of target with the initial range $r_{i}$ and constant radial velocity $v_{j}$.

Finally, go through all the searching parameters, we can get a two-dimensional result defined in the range-velocity plane, which is the result of coherent integration based on Radon-NUFFT method.

**Figure 2:**
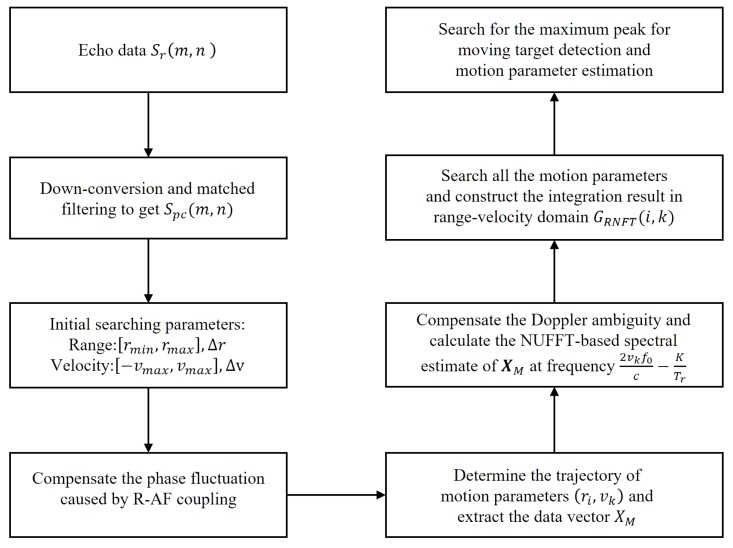
Flow chart of the proposed method.

According to the analysis above, when the search parameters are equal to the real motion parameters of target, the RCM and the phase fluctuations caused by R-AF coupling and V-T-AF coupling can be compensated accordingly, so that the target energy can be fully accumulated. After that, the detection of moving target and estimation of motion parameters can be achieved by peak searching. The flow chart of the coherent integration method based on Radon-NUFFT for FA radar is shown in Figure 2.

## 4. Simulation Results

In this section, some results of simulation experiments are presented to validate the performance of the coherent integration method based on Radon-NUFFT. The simulated parameters of the radar system and the moving target are listed in Table 1.

**Figure 3:**
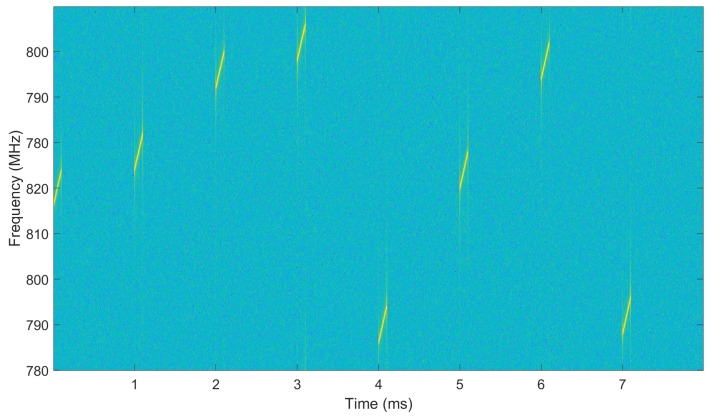
Spectrogram of transmitted signal.

Figure 3 shows the spectrogram of the transmitted signal of the simulated radar system. It can be seen that the carrier frequency of each pulse hops randomly between different frequency.

**Figure 4:**
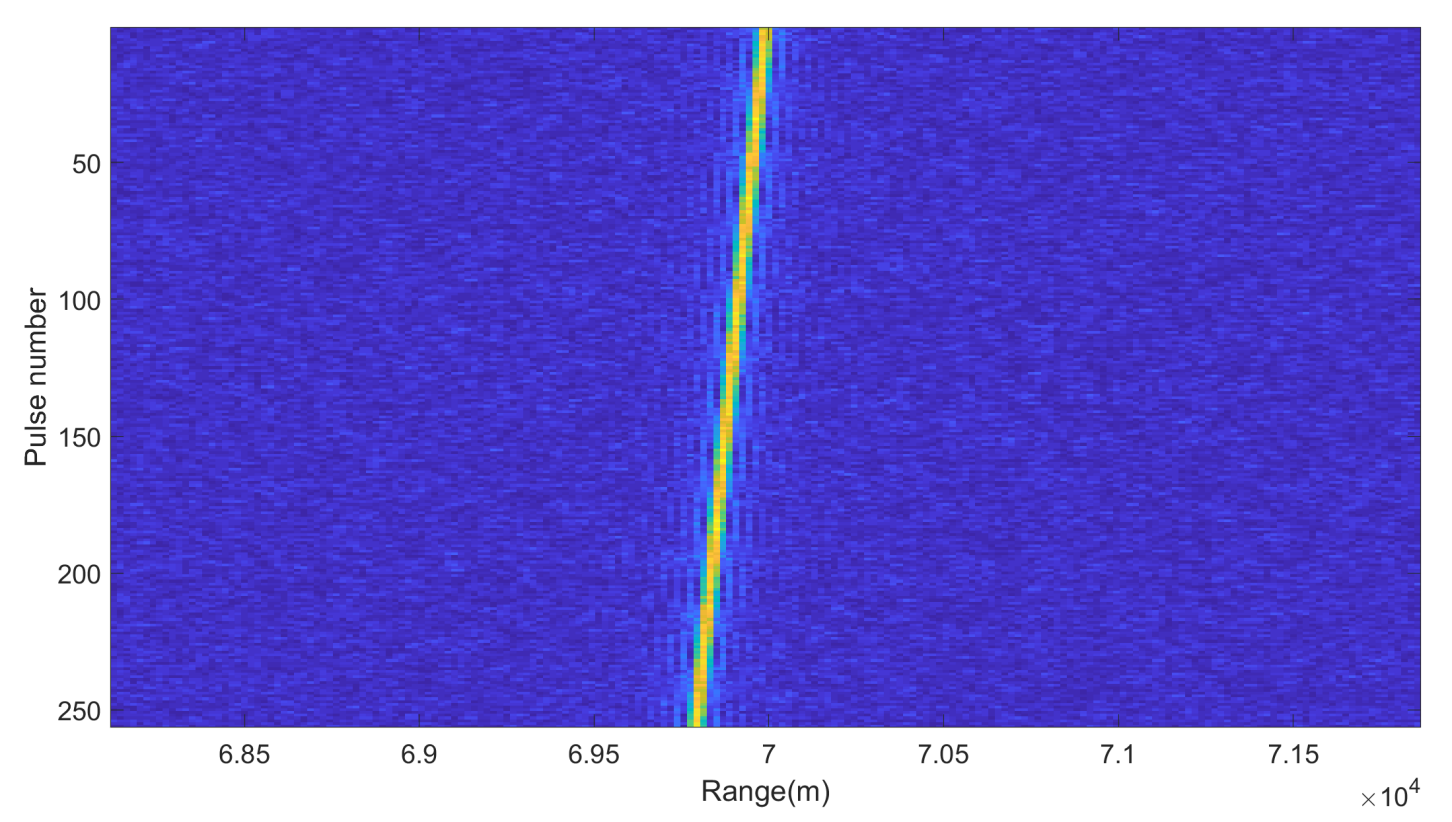
Range-time map after pulse compression.

Suppose the SNR of the received target echo is −10 dB, after the down-conversion and pulse compression of 256 echo signal, the time-range map can be rearranged as shown in Figure 4. It can be seen that the trajectory of the target is an oblique line due to the RCM.

**Figure 5:**
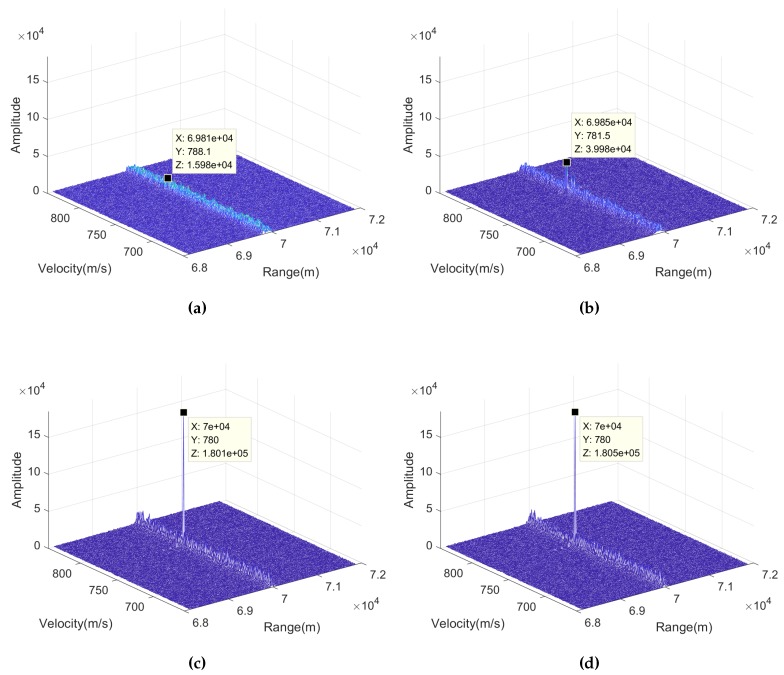
Coherent integration via four method: (a) MTD, (b) NUFFT, (c) FA-CRT, (d) Radon-NUFFT.

After that, Figure 5 compares the results of coherent integration via four methods. Figure 5a is the result of conventional moving target detection (MTD), i.e., perform FFT directly in the slow time dimension after compensation of the phase fluctuation caused by R-AF coupling. Figure 5b is the result of performing NUFFT among the slow time dimension after compensation of the phase fluctuation caused by R-AF coupling, which is denoted as NUFFT for brevity. Figure 5c is the result of FA-CRT, which compensate all the phase fluctuations directly while searching motion parameters. In addition, Figure 5d is the result of Radon-NUFFT.

It can be seen that the positions of the peaks and the corresponding values in Figure 5 are marked, so that Table 2 can be obtained as follows.

It can be seen that since MTD cannot deal with the problem of RCM and nonuniform phase fluctuation caused by R-AF coupling, it cannot obtain an apparent peak in the range–velocity plane, and the estimated parameters of the target are inaccurate. As for the result of NUFFT, since the method cannot compensate the RCM, the estimated range and velocity of target has a small deviation from the real parameters, and the peak value is lower than that in Radon-NUFFT and FA-CRT due to the energy dispersion caused by RCM. Comparing the results of Radon-NUFFT and FA-CRT, it can be seen that the two results are similar, both of them can get accurate target motion parameters, and also can fully accumulate target energy. Because they are based on the same principle, which can compensate the RCM and phase fluctuations caused by R-AF coupling and V-T-AF coupling. The differences between the two methods is the implementation process, which is related with the operation speed that we will discuss it later.

**Figure 6:**
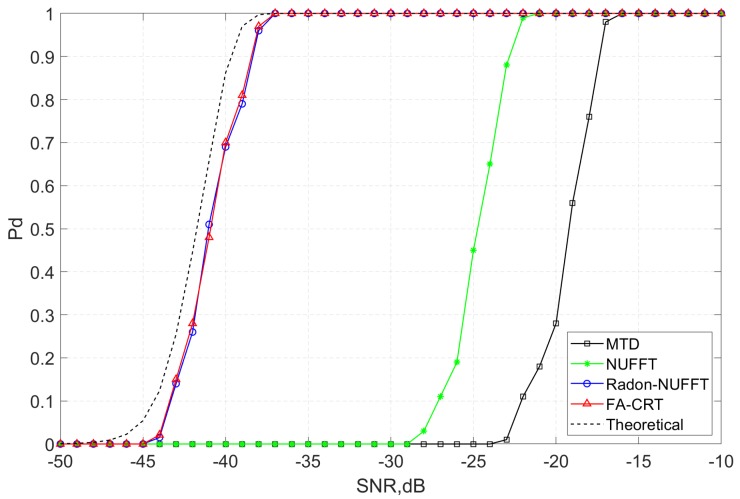
Detection probability of MTD, NUFFT, FA-CRT, Radon-NUFFT and theoretical coherent integration.

To quantitatively analyze the detection performance of the proposed method, we perform Monte Carlo experiments to compare the detection probability of MTD, NUFFT, FA-CRT and Radon-NUFFT. Set the range of SNR of the echo signal before pulse compression as −50 to −10 dB, and the false alarm rate is set as $P_{fa}=10^{-6}$. The cell-averaging constant false alarm rate (CA-CFAR) detector is applied to detect the target in the coherent integration result, and corresponding detection probability $P_{d}$ is calculated through Monte Carlo trials. In addition, the detection probability of the theoretical coherent integration result is calculated by the Marcum Q function [24]. Figure 6 shows the detection probability of theoretical coherent integration and the four methods again different SNR levels.

As shown in Figure 6, the detection probability of MTD will decline sharply when SNR < −16 dB since MTD cannot deal with RCM and nonuniform phase fluctuation caused by R-AF coupling. The detection performance of NUFFT is better than MTD, and the detection probability of Radon-NUFFT and FA-CRT are almost the same. According to the simulated parameters in Table 1, the theoretical coherent integration gain is 24 dB. For the same detection probability $P_{d}=0.7$, the required SNR of theoretical coherent integration, Radon-NUFFT, FA-CRT, NUFFT, and MTD are −40.8, −40, −40, −23.8 and −18.3 dB, which indicates that the integration gain for the compensation of RCM is 16.2 dB, the integration gain for the compensation of phase fluctuation caused by V-T-AF coupling is 5.5 dB, and the coherent integration gain of the proposed method is only 0.8 dB lower than the theoretical coherent integration gain.

Finally, we can analyze the computational complexity of the four methods by comparing the operation time of four methods for one trail, with the searching scope of range is set as [67, 73] km and the searching scope of velocity is [500, 1000] m/s, the result of computing time is shown in Table 3. The main configuration of the platform is as follows: CPU: Intel(R) Core(TM) i7-6600U CPU @2.6GHz 2.81GHz; RAM: 12 GB; operating system: Windows 10; software: Matlab 2018a.

As shown in Table 3, MTD takes the least amount of time since it can be easily achieved by FFT among the slow-time dimension. In addition, NUFFT takes the second least amount of time, because the NUFFT based on low rank approximation can be implemented by several FFTs. It is obvious that Radon-NUFFT takes less time than FA-CRT, since FA-CRT compensate all the phase fluctuations directly while searching motion parameters, which requires abundant computation, while Radon-NUFFT uses NUFFT based on low rank approximation to realize the compensation of phase fluctuation caused by velocity-time-AF quickly and accurately.

## 5. Conclusions

In this paper, we proposed a coherent integration method based on Radon-NUFFT for moving target detection using an FA radar. The method combined Radon algorithm with NUFFT based on low rank approximation to compensate the RCM caused by target motion and nonuniform phase fluctuations among different pulses caused by R-AF coupling and V-T-AF coupling, so that the target energy can be fully accumulated. The coherent integration result was defined in the range-velocity plane, so the target detection and estimation of motion parameters can be achieved by peak searching afterwards. Finally, simulation experiments were conducted to demonstrate the effectiveness of the proposed method, and the results showed that the proposed method is superior to the MTD and NUFFT in terms of detection probability and estimation accuracy, and the performance of computational efficiency is better than FA-CRT. A possible future research work might concern the implementations of the Radon-NUFFT in real engineering applications.

## Figures and Tables

**Table 1:** Simulated parameters.

Simulated Parameters (Unit)	Values
Average carrier frequency (MHz)	800
Variation range of carrier frequency (MHz)	[780,820]
Variation interval of carrier frequency (MHz)	1
Sampling frequency (MHz)	8
Bandwidth (MHz)	4
Pulse width (us)	100
Pulse repetition interval(us)	1000
Number of coherently integrated pulses	256
Initial distance of target (km)	70
Radial velocity of target (m/s)	780

**Table 2:** Location and value of the maximum peak points.

Method	Range (m)	Velocity (m/s)	Amplitude
MTD	$6.981\times 10^{4}$	788.1	$1.598\times 10^{4}$
NUFFT	$6.985\times 10^{4}$	781.5	$3.998\times 10^{4}$
FA-CRT	$7\times 10^{4}$	780	$1.801\times 10^{5}$
Radon-NUFFT	$7\times 10^{4}$	780	$1.805\times 10^{5}$

**Table 3:** Computing time of the four methods.

Method	MTD	NUFFT	Radon-NUFFT	FA-CRT
Computing time(s)	0.003796	0.036970	0.684318	6.105591
